# Endoscopic removal of a nasogastric tube inadvertently stapled to the gastric stump

**DOI:** 10.1055/a-2891-5827

**Published:** 2026-07-08

**Authors:** Shibo Song, Bin Zhou, Han Yan, Long Rong

**Affiliations:** 1 Endoscopy Center 26447 Peking University First Hospital, Beijing, Beijing, China

## A case description



**Video 1** Endoscopic retrieval of an entrapped nasogastric tube (NGT) 1 month postoperatively.


**Fig. 1:**
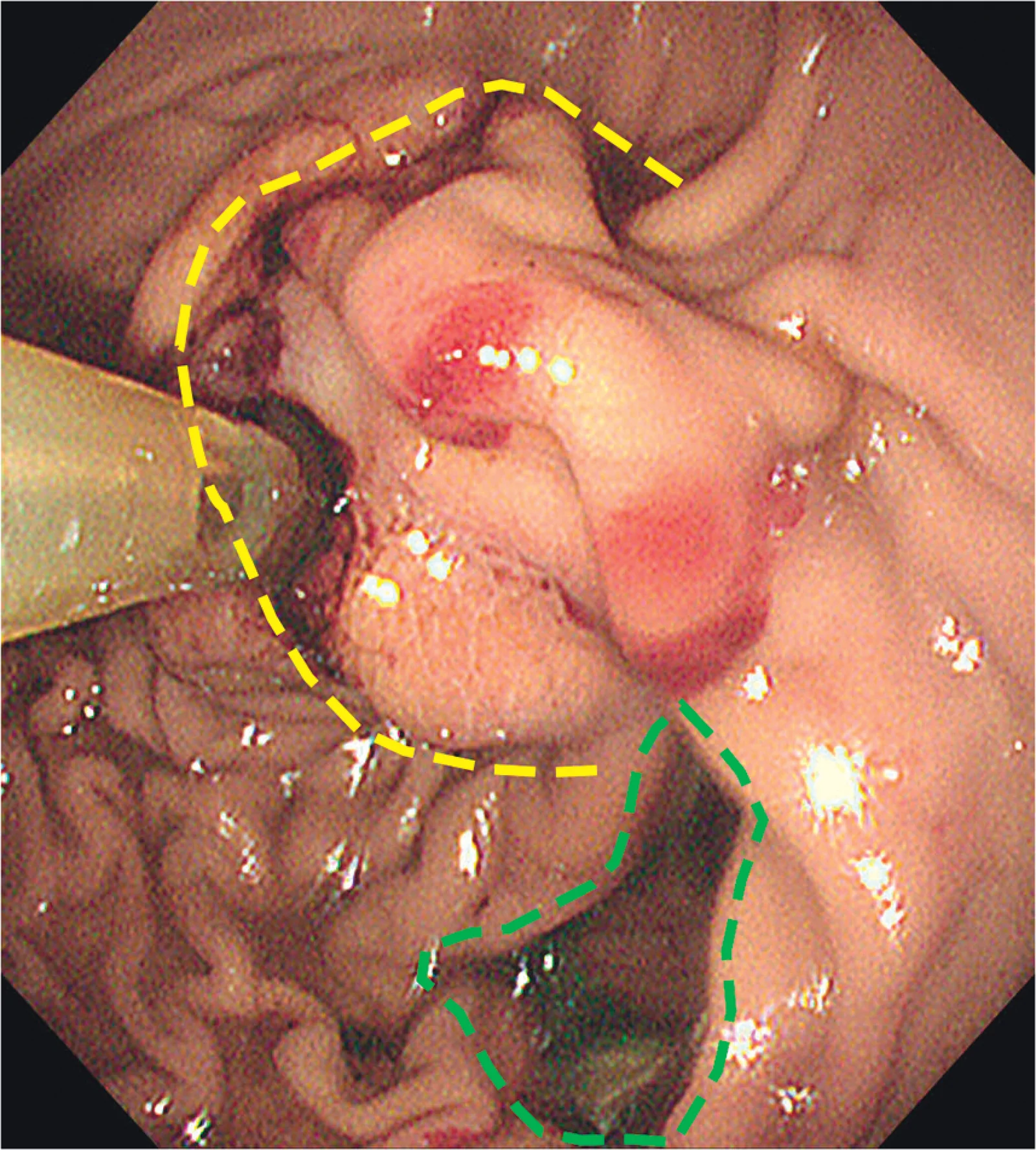
An endoscopic view on postoperative day 3: The nasogastric tube is stapled to the gastric stump staple line. Note: The yellow dashed line is the gastric stump staple line and the green dashed line is the gastrojejunal anastomosis **.**

A 65-year-old man underwent a Whipple procedure for biliary malignancy. On postoperative day 3, gastroscopy for decreasing hemoglobin revealed that the tip of the nasogastric tube (NGT; DRW-B, 14F; Dare, Baoji, China) was inadvertently incorporated into the staple line of the gastric stump, near the gastrojejunostomy ( **Figs.** 1 **and** 2 ). The tube was found to be fixed, with no staples visible under endoscopy ( Fig. 3 ). Notably, three proximal side holes were identified within the gastric lumen, maintaining their functional patency. To mitigate the risk of a postoperative fistula from forced extraction, a multidisciplinary team opted for 1 month of conservative management. During this period, the patient gradually advanced to liquids. Although intermittent mild abdominal pain and distension occurred, these symptoms remained tolerable with oral acid suppressants and prokinetics.

**Fig. 2:**
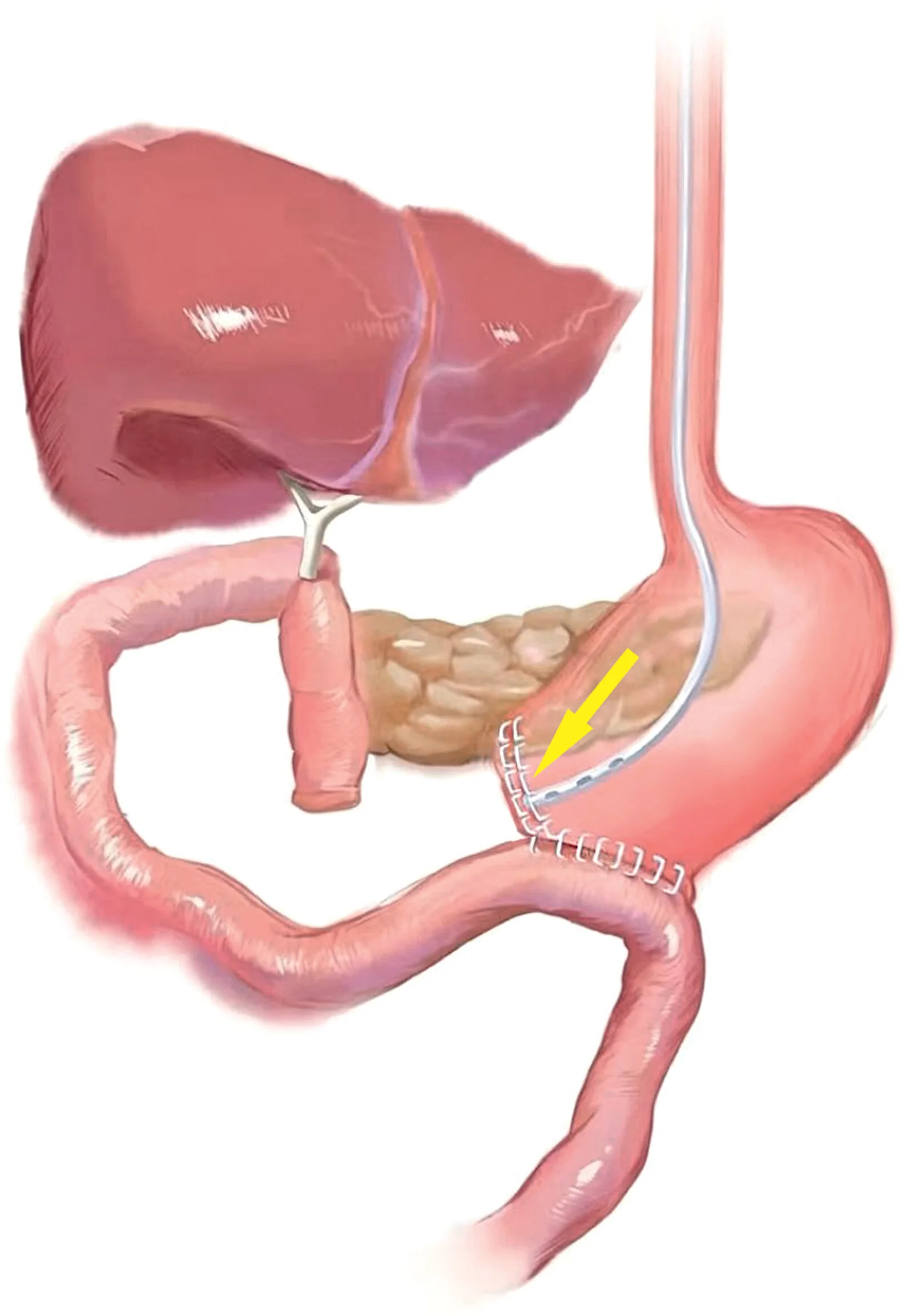
Schematic illustration of the nasogastric tube position relative to the surgical anatomy. Note: The yellow arrow indicates the entrapment site within the gastric stump staple line. Illustration credit: This illustration was prepared by our professional illustrator, Meishen Liu (PhD Candidate in Architecture, MFA, Beijing University of Civil Engineering and Architecture), with expertise in professional drawing.

**Fig. 3:**
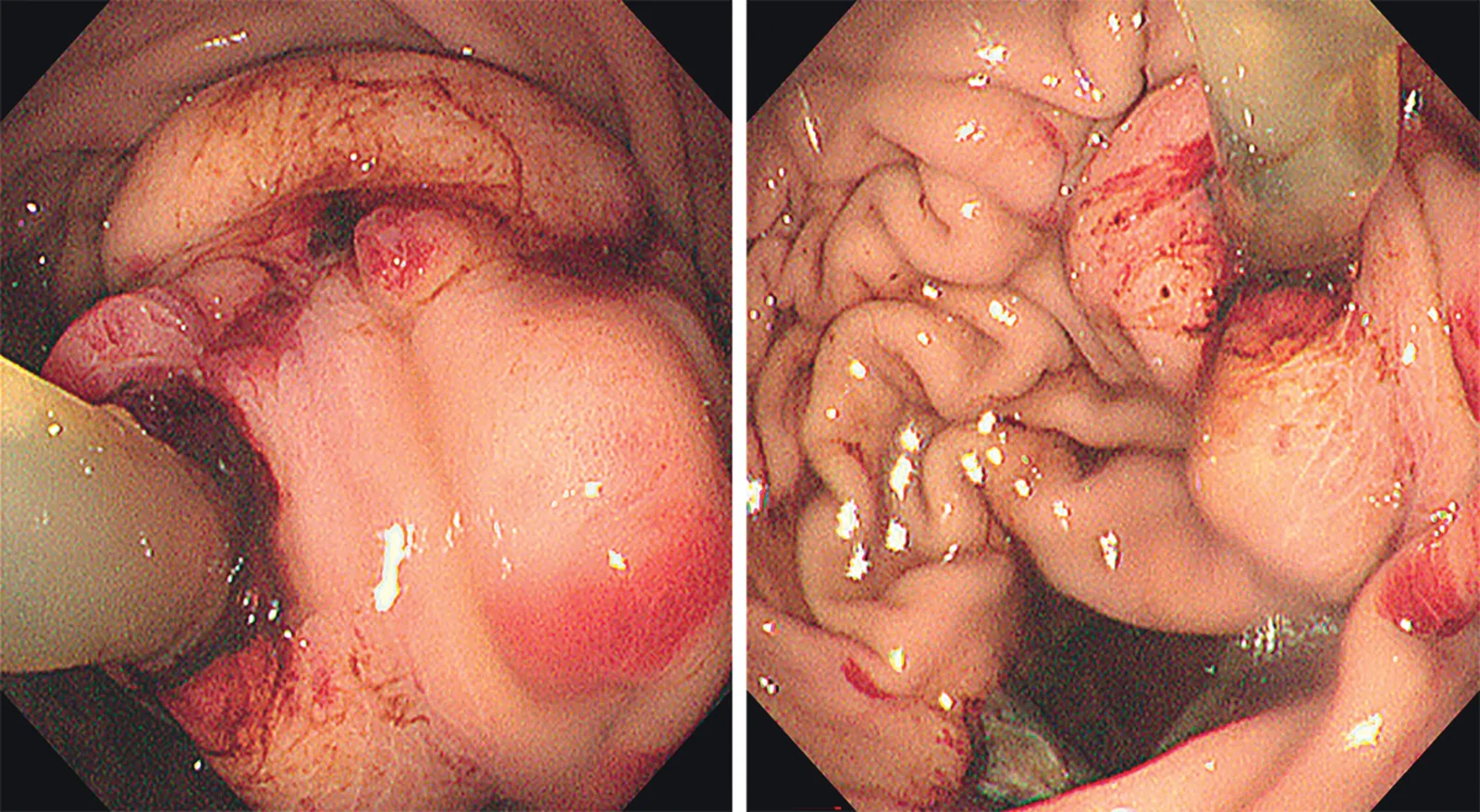
An endoscopic view of the fixed nasogastric tube with no visible staples.

**Fig. 4:**
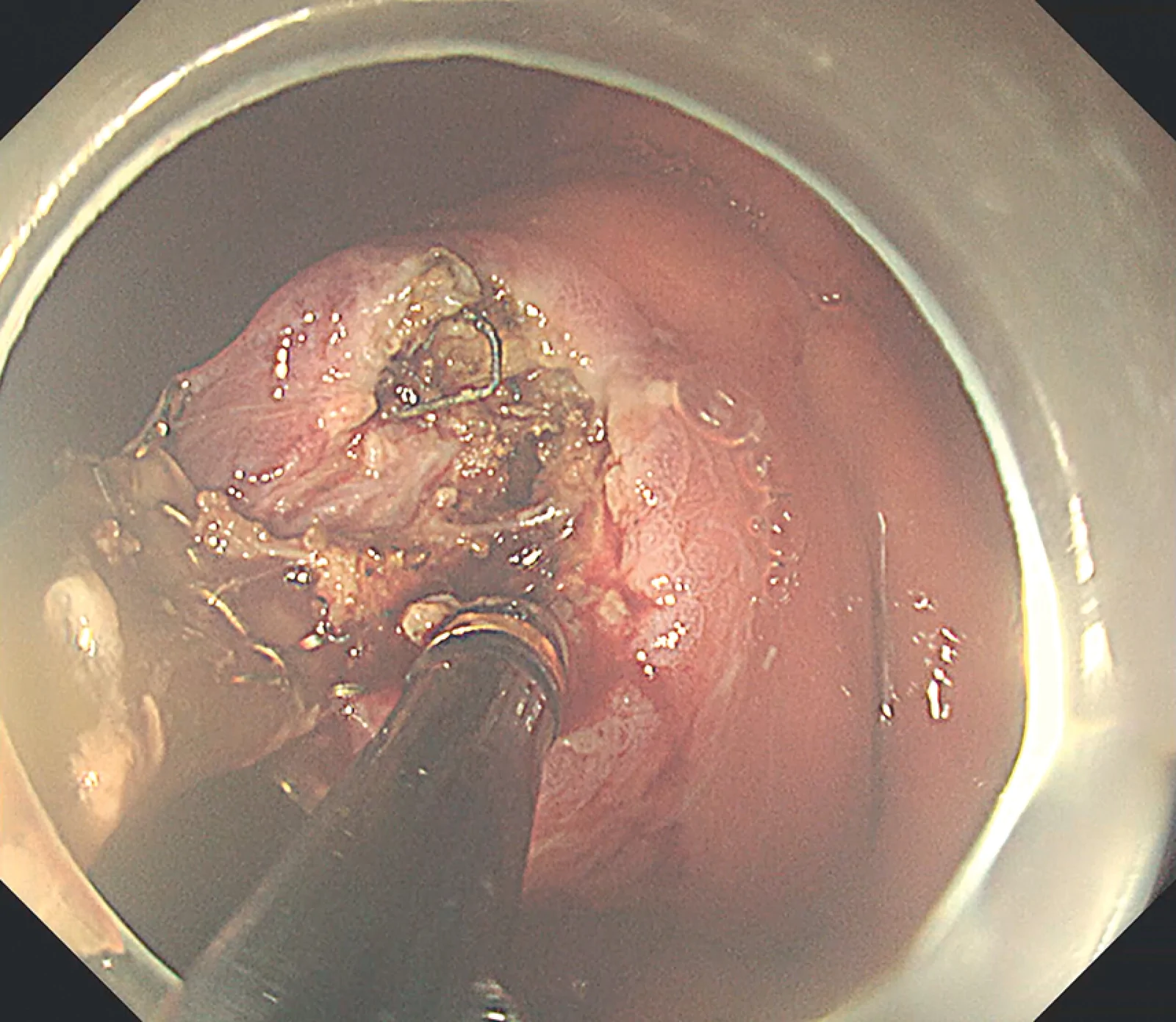
Endoscopic incision of the tissue surrounding the entrapped NGT using an electrosurgical knife.

**Fig. 5:**
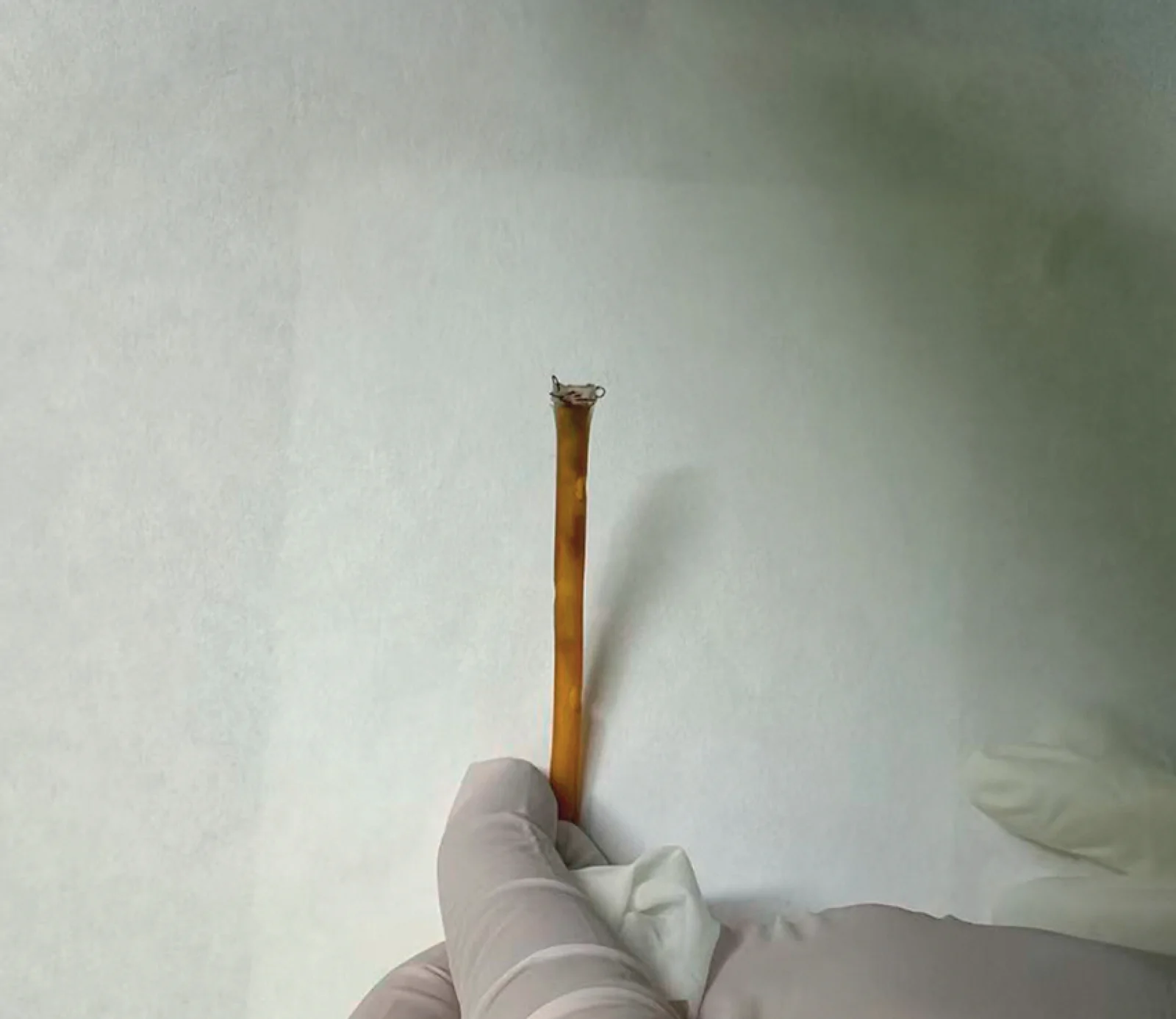
The extracted NGT showing a neatly transected end with three rows of embedded surgical staples.

One month later, under general anesthesia, an endoscopic incision of the mucosa and muscularis propria surrounding the entrapped NGT was performed with an electrosurgical knife (MK-T-1-195; Micro-Tech, Nanjing, China; Fig. 4 and Video 1 ). The NGT was then easily extracted, revealing a neatly transected end with three rows of embedded surgical staples—a clear indication of stapler transection that rendered manual withdrawal impossible ( Fig. 5 ). The 15-minute procedure was completed without perforation, and the defect was closed with three clips. Pathological review confirmed that the severed distal NGT segment was retrieved within the original surgical specimen, and follow-up computed tomography verified the absence of any residual fragments. The patient was discharged uneventfully 3 days later.

Intraoperative NGT entrapment is rare, typically involving sutures rather than surgical staples. ^1^ A previous report suggests cutting the tube, but this often leaves foreign fragments behind. ^2^ Our delayed strategy ensures safe and intact NGT retrieval via precise endoscopic incision after sufficient tissue healing. This strategy represents a secure and effective alternative for managing NGTs entrapped in surgical staple lines.

Endoscopy_UCTN_Code_TTT_1AO_2AN
